# Untargeted Metabolomic Differences Between Colostrum and Mature Milk in Lactating Sows: Biological Significance and Implications

**DOI:** 10.3390/ani16213504

**Published:** 2026-11-06

**Authors:** Diego Amaya, Jhones O. Sarturi, Janghan Choi

**Affiliations:** Department of Animal and Food Sciences, Texas Tech University, Lubbock, TX 79409, USA; damayacr@ttu.edu (D.A.); j.sarturi@ttu.edu (J.O.S.)

**Keywords:** colostrum, milk, lipids, sows, untargeted metabolomics

## Abstract

This study investigated the temporal metabolic differences between sow colostrum and mature milk using an untargeted metabolomics approach. Nine multiparous Camborough sows were longitudinally sampled for colostrum and mature milk collection. Proximate analysis demonstrated that protein content was significantly greater in colostrum than in mature milk (*p* < 0.05). Untargeted metabolomic profiling successfully putatively annotated 3906 metabolites, with 560 significantly increased and 1083 decreased in colostrum compared to mature milk. Multivariate statistical analyses, including PCA and OPLS-DA, confirmed distinct metabolic separation between the two lactation stages. Furthermore, pathway enrichment and Differential Abundance score analyses revealed that diverse lipid metabolism pathways, fat digestion and absorption, thermogenesis, and glycerolipid synthesis were significantly elevated in mature milk. Physicochemical evaluation indicated that mature-milk-enriched metabolites exhibited significantly greater molecular weights and Log P values, reflecting enhanced structural complexity and lipophilicity. These comprehensive differences suggest that mature milk features higher-molecular-weight and lipophilic metabolites aligned with changing nutritional requirements during later lactation, whereas colostrum is enriched with simpler fractions characteristic of the early postpartum stage. Collectively, this study establishes a comprehensive comparative metabolic baseline of sow milk, providing a robust framework for understanding maternal metabolite dynamics with potential application for optimizing sow nutrition and milk quality.

## Simple Summary

Sow milk naturally transforms between early and established lactation to meet the evolving nutritional needs of growing piglets. The first milk produced after birth, known as colostrum, is vital because it provides easily absorbed nutrients and biological compounds that help newborns survive and adapt to life outside the womb. However, the detailed chemical differences between colostrum and later-stage milk have not been fully clear. In this study, we compared colostrum collected between 12 and 24 h after farrowing with mature milk collected on day 28 of lactation, identifying thousands of distinct compounds. Our findings showed that colostrum is packed with simpler, smaller molecules designed for quick and direct support during the first days of life. In contrast, mature milk contains a richer variety of more complex, larger fat-related molecules that support the steady growth and development of older piglets. Overall, this research maps out the distinct nutritional shifts in sow milk, providing a clearer picture of how mothers nourish their young and offering a foundation for improving future swine nutrition and piglet health.

## 1. Introduction

Swine production is a highly complex system in which each production phase presents distinct biological and management challenges [1]. Among these, the lactation period immediately after farrowing is particularly critical for piglet survival and subsequent performance [2]. During this early postnatal stage, newborn piglets must rapidly adapt to extrauterine life while relying almost entirely on sow milk as their sole source of nutrients, bioactive compounds, and immune protection. Despite advances in modern swine production systems, pre-weaning mortality remains substantial, often reaching approximately 10–20%, underscoring the importance of early-life nutritional and immunological support for improving survival and post-weaning growth performance [3].

Sow colostrum and milk provide essential macronutrients alongside a diverse array of bioactive components including immunoglobulins, growth factors, and metabolites that are critical for neonatal growth and development [4,5]. Colostrum serves as the primary vehicle for passive immunity transfer and early metabolic programming, making it indispensable for piglet health and development [6]. In contrast, mature milk reflects a more stable nutritional matrix that supports sustained growth and physiological maintenance. Tan et al. [7] suggested clear metabolomic differences between sows with high and low lactation performance. Although the importance of colostrum and mature milk composition has been well established [4], the majority of previous studies have focused on targeted nutrient analysis or limited metabolite profiling [8,9].

Consequently, a comprehensive understanding of the global metabolic landscape comparing colostrum and mature milk remains incomplete. Recent advances in metabolomics have enabled broader characterization of biological samples including diverse animal-derived matrices such as meat products (e.g., pork, beef, and poultry), intestinal and gut-related samples, and other biological fluids and tissues [10,11,12,13]. Previous studies by Curtasu et al. [9], Tan et al. [7], and Settachaimongkon et al. [14] provided valuable insights into the lipidomic and metabolic compositions of colostrum and milk, but they were constrained by relatively limited metabolite coverage and analytical resolution. To address these limitations, the current study utilizes high-resolution untargeted metabolomics to deliver a significantly broader metabolite coverage and higher analytical depth. Therefore, the objective of this study was to investigate the metabolic differences between colostrum and mature milk in sows using an untargeted metabolomics approach.

## 2. Materials and Methods

### 2.1. Animals and Experimental Design

A total of 9 lactating PIC Camborough sows (Pig Improvement Company, Hendersonville, TN, USA) with parities ranging from 3 to 8 were used in this study (Average: 5.1). The same sows were sampled longitudinally for both colostrum and mature milk collection. Colostrum samples were collected on d 1 of lactation, and mature milk samples were collected on d 28 of lactation (averaging d 25.8 postpartum across sows due to a 1–2 day variation in actual farrowing dates) prior to weaning. All sows were housed individually in farrowing crates under standard commercial management conditions. Table 1 shows individual sow information used in the study. Detailed characteristics regarding parity, pre- and post-farrowing body weight changes, lactation duration, and litter performance traits are summarized. All sows were clinically healthy and received no therapeutic medication during the experimental period.

All animals were fed a common gestation and lactation diet formulated to meet or exceed the nutrient requirements recommended by the NRC (2012) for swine [15] (Table 2). During gestation, sows were fed the gestation diet to maintain a body condition score (BCS) of 3. Upon farrowing, lactation diets were provided, and feed allowance was adjusted using a farm-managed step-up feeding program throughout the lactation period. Water was provided ad libitum throughout the entire experimental period. All experimental procedures were reviewed and approved by the Texas Tech University Institutional Animal Care and Use Committee (IACUC; protocol 2025-1666). Sample collection was conducted in the morning. Colostrum samples were collected between 12 and 24 h after farrowing, whereas mature milk samples were collected on d 28 of lactation prior to weaning. Prior to sample collection, each sow received an intramuscular injection of 1 mL oxytocin (20 USP units/mL; Bimeda, Le Sueur, MN) in the neck region to stimulate milk letdown [16]. Piglets were temporarily relocated to adjacent pens during the collection procedure. Approximately 5 min after administration, colostrum or mature milk was manually collected from the first five functional teats in a cranial-to-caudal direction on the right side of the mammary chain over a 15-min period using sterile gloves and sterile collection tubes to minimize contamination, and equal volumes from each teat were pooled into a composite sample for each sow. A total of 100 mL of sample was collected per sow. Samples were immediately placed on ice and transported to the laboratory within 30 min of collection. Upon arrival, samples were aliquoted into sterile 1.5-mL microcentrifuge tubes and stored at −80 °C until metabolomic analysis. A total of 18 samples (9 sows × 2 time points: colostrum and mature milk) were collected for metabolomic analysis.

### 2.2. Milk Composition Analysis

Colostrum and mature milk samples (50 mL per sample) were submitted to Eastern Laboratory Services (Medina, OH, USA) for compositional analysis. Fat content was determined using ether extraction according to AOAC method 989.05, and protein content was analyzed according to AOAC method 991.20. Fat and protein concentrations were expressed as percentages (%).

### 2.3. Metabolomic Analysis

Untargeted metabolomics analysis was conducted by Metware Biotechnology (Woburn, MA, USA). Samples stored at −80 °C were thawed on ice and vortexed for 10 s prior to analysis. Briefly, 50 μL of each sample was transferred into a 2 mL microcentrifuge tube and mixed with 300 μL of extraction solvent (acetonitrile:methanol, 1:4, *v*/*v*) containing internal standards (L-phenylalanine-d5 [MWS3085-IS-P, Q1=281.006 Da, RT=4.31 min], L-leucine-d3 [MWS04113-IS-P, Q1=139.14 Da, RT=1.26 min], L-tryptophan-d5 [MWS20572-IS-P, Q1=198.09 Da, RT=3.08 min], and L-methionine-d3 [MWS04187-IS-P, Q1=210.13 Da, RT=2.34 min]). Internal standards with known concentrations were added to the quality control (QC) samples for assessing variations between samples, demonstrating high analytical reproducibility with coefficients of variation (CV) ranging from 0.009 to 0.02. A pooled QC sample was prepared by mixing equal aliquots (10 μL) from each extracted colostrum and mature milk sample to represent the entire sample matrix and monitor the overall analytical stability. The mixture was vortexed for 3 min and centrifuged at 15,313× *g* for 10 min at 4 °C using a refrigerated centrifuge (5430R; Eppendorf, Hamburg, Germany). Subsequently, 200 μL of the supernatant was transferred to a new tube and incubated at −20 °C for 30 min to enhance protein precipitation, followed by centrifugation at 15,313× *g* for 3 min at 4 °C. Finally, 180 μL of the supernatant was collected and used for LC–MS analysis. Metabolomic profiling was performed using an ultra-performance liquid chromatography system (ExionLC AD; SCIEX, Redwood City, CA, USA) coupled with a tandem mass spectrometer (TripleTOF 6600+; SCIEX, Redwood City, CA, USA). For reversed-phase separation, metabolites were analyzed using an Acquity UPLC HSS T3 column (1.8 μm, 2.1 × 100 mm; Waters, Milford, MA, USA). The mobile phases consisted of ultrapure water containing 0.1% formic acid (mobile phase A) and acetonitrile containing 0.1% formic acid (mobile phase B). The T3 elution gradient was programmed as follows: 0 min, 95% A (5% B); 2 min, 80% A (20% B); 5 min, 40% A (60% B); 6–7.5 min, 1% A (99% B); 7.6–10 min, re-equilibrated at 95% A (5% B). The column temperature was maintained at 40 °C with a flow rate of 0.4 mL/min and an injection volume of 4 μL. For hydrophilic interaction liquid chromatography (HILIC) analysis, separation was performed using an ACQUITY Premier BEH Amide column (1.7 μm, 2.1 × 150 mm; Waters, Milford, MA, USA). Mobile phase A consisted of 60% acetonitrile, 30% water, and 10% methanol containing 20 mM ammonium formate (pH 10.6), while mobile phase B consisted of 40% acetonitrile and 60% water containing 20 mM ammonium formate (pH 10.6) [17]. The HILIC gradient was set as follows: 0 min, 95% A (5% B); 3.5 min, 70% A (30% B); 5.5–6.5 min, 5% A (95% B); 6.51–10 min, re-equilibrated at 95% A (5% B). The column temperature was maintained at 40 °C with a flow rate of 0.4 mL/min and an injection volume of 4 μL throughout the analysis.

Mass spectrometry acquisition was operated in both positive (ESI+) and negative (ESI−) electrospray ionization modes over a total run duration of 10 min. ESI parameters were configured as follows: IonSpray Voltage, 5000 V (ESI+)/−4000 V (ESI−); Source Temperature, 550 °C (ESI+)/450 °C (ESI−); Ion Source Gas 1 (GS1), 50 psi; Ion Source Gas 2 (GS2), 60 psi; Curtain Gas (CUR), 35 psi; and Declustering Potential (DP), ±60 V. MS1 TOF mass range was set to m/z 50–1000 Da with an accumulation time of 0.2 s and collision energy (CE) of ±10 V. MS2 TOF mass range was set to *m*/*z* 25–1000 Da with an accumulation time of 0.04 s, CE of ±30 V, and CE spread of 15 V. The top 18 candidate ions per cycle were selected for fragmentation with dynamic exclusion (excluded for 3 s after 3 occurrences).

### 2.4. Data Processing, Multivariate, and Enrichment Analyses

Metabolomic data processing, multivariate analyses, and pathway enrichment analyses were conducted following procedures previously described by Choi et al. [10] with modifications appropriate for the current study design. Raw UPLC–MS data files were converted into mzXML format using ProteoWizard prior to data processing. Peak detection, retention time alignment, and normalization were performed using the XCMS package in R software. Features with more than 50% missing values within either colostrum or mature milk samples were excluded from further analysis [18]. Remaining missing values were imputed using the k-nearest neighbor (KNN) algorithm, and signal drift correction was performed using support vector regression (SVR). The blank values were filled with KNN and 1/5 minimum (1/5 minimum filled for blank values > 50%, KNN filled for blank values < 50%), and the peak area was corrected by SVR method. Metabolite annotation was conducted by matching detected features against MetwareBio’s in-house library, public metabolite databases, prediction databases, and metDNA. Metabolites were annotated by matching detected features against MetwareBio’s in-house library, public metabolite databases, prediction databases, and metDNA, based on accurate mass (±10 to 20 ppm), MS/MS spectral similarity, and isotopic distribution. Metabolites with a comprehensive identification score > 0.7 and a coefficient of variation (CV) < 0.3 in quality control (QC) samples were retained for downstream analyses [19]. Positive and negative ionization datasets were merged by retaining metabolites with the highest identification confidence and lowest CV values. Multivariate statistical analyses were performed using R software (version 4.1.2). Principal component analysis (PCA) with unit variance scaling was performed to assess the overall distribution and clustering of colostrum and mature milk samples. Orthogonal partial least squares–discriminant analysis (OPLS-DA) was subsequently conducted using the MetaboAnalystR package to maximize group separation and identify metabolites contributing to the observed differences. The robustness and predictive ability of the OPLS-DA model were evaluated using R^2^Y and Q^2^ values. Differential metabolites between the colostrum and mature milk were selected based on a variable importance in projection (VIP) score > 1 and a *p* value < 0.05. For all statistical analyses, *p* < 0.05 was defined as the threshold for statistical significance. The VIP score reflects the contribution of each metabolite to group discrimination in the OPLS-DA model with metabolites exhibiting VIP > 1 considered important contributors to the separation between the two groups. Volcano plots were constructed using log2 fold changes and −log10 transformed *p*-values to visualize the overall distribution and significance of metabolite changes. Hierarchical clustering analysis was further performed based on the relative abundance patterns of significantly altered metabolites across samples. Three-dimensional principal component analysis (3D PCA) was performed to visualize the overall variation and clustering patterns among samples. PC1, PC2, and PC3 represent the first, second, and third principal components, respectively. The percentage associated with each principal component indicates the proportion of total variance explained by that component. To further characterize metabolic differences, the top 15 differentially expressed metabolites were ranked according to log2 fold change values between colostrum and mature milk samples. Metabolites with increased and decreased levels were evaluated to identify those showing the greatest abundance differences between the two lactation stages. Pearson correlation analysis was performed to assess relationships among differentially abundant metabolites putatively annotated using the previously described screening criteria. Pathway enrichment analysis of significant metabolites (*p* < 0.05) was performed using the Kyoto Encyclopedia of Genes and Genomes (KEGG) database to identify biological pathways associated with metabolic alterations between colostrum and mature milk. The hypergeometric test was applied to determine whether differential metabolites were significantly enriched in specific metabolic pathways, and P values were adjusted for multiple testing using the False Discovery Rate (FDR) method. KEGG DA scores were calculated to evaluate the overall direction of metabolic changes within each pathway using the following formula:

$$\mathrm{DA}\;\mathrm{score}=\frac{\mathrm{Number}\;\mathrm{of}\;\mathrm{increased}\;\mathrm{metabolites}-\mathrm{Number}\;\mathrm{of}\;\mathrm{decreased}\;\mathrm{metabolites}}{\mathrm{Total}\;\mathrm{number}\;\mathrm{of}\;\mathrm{annotated}\;\mathrm{metabolites}\;\mathrm{in}\;\mathrm{the}\;\mathrm{pathway}}$$

The molecular weights and octanol–water partition coefficients (Log P) of the differential metabolites of colostrum and mature milk were retrieved from PubChem using Python (v3.11; Python Software Foundation) for an unweighted comparison of physicochemical properties between the two lactation stages.

### 2.5. Statistical Analysis

Milk composition data were evaluated using a two-tailed paired Student’s *t*-test, while molecular weights and Log P (logarithm of the octanol–water partition coefficient) values between the increased and decreased metabolite groups were compared using a two-tailed unpaired Student’s *t*-test. All corresponding statistical analyses were performed using GraphPad Prism (version 11.0.1; GraphPad Software, San Diego, CA, USA). For untargeted metabolomic data analyzed using R software packages, differential metabolites between colostrum and mature milk were identified using a combination of orthogonal partial least squares–discriminant analysis (OPLS-DA) with a variable importance in projection (VIP) score > 1 and a two-tailed paired Student’s *t*-test yielding (*p* < 0.05). Statistical significance for all other evaluations was defined at (*p* < 0.05).

## 3. Results

### 3.1. Milk Composition

**Figure 1:**
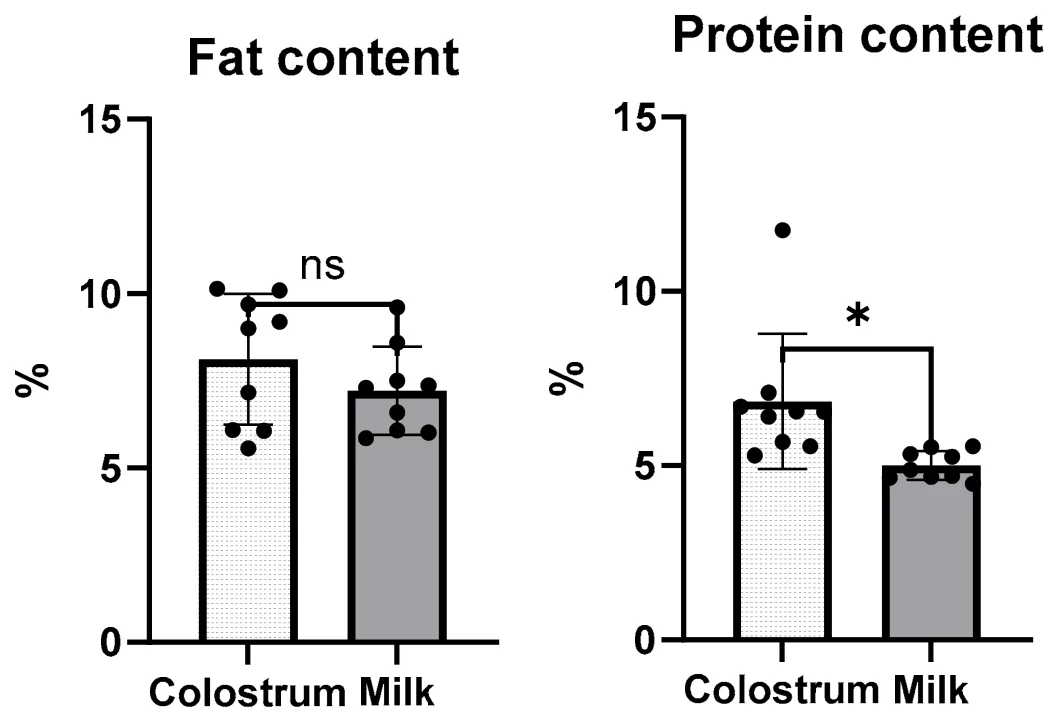
Fat and protein of colostrum and mature milk in lactating sows. ns: non-significant; * *p* < 0.05. Values represent means ± SEM.

Figure 1 shows the comparison of colostrum (d 1) and mature milk (d 28) composition. No differences were observed in fat content between colostrum and mature milk (*p* = 0.25). However, colostrum contained significantly higher protein content compared with mature milk (*p* < 0.05).

### 3.2. Differential Metabolomic Profiles and Volcano Plots

**Figure 2:**
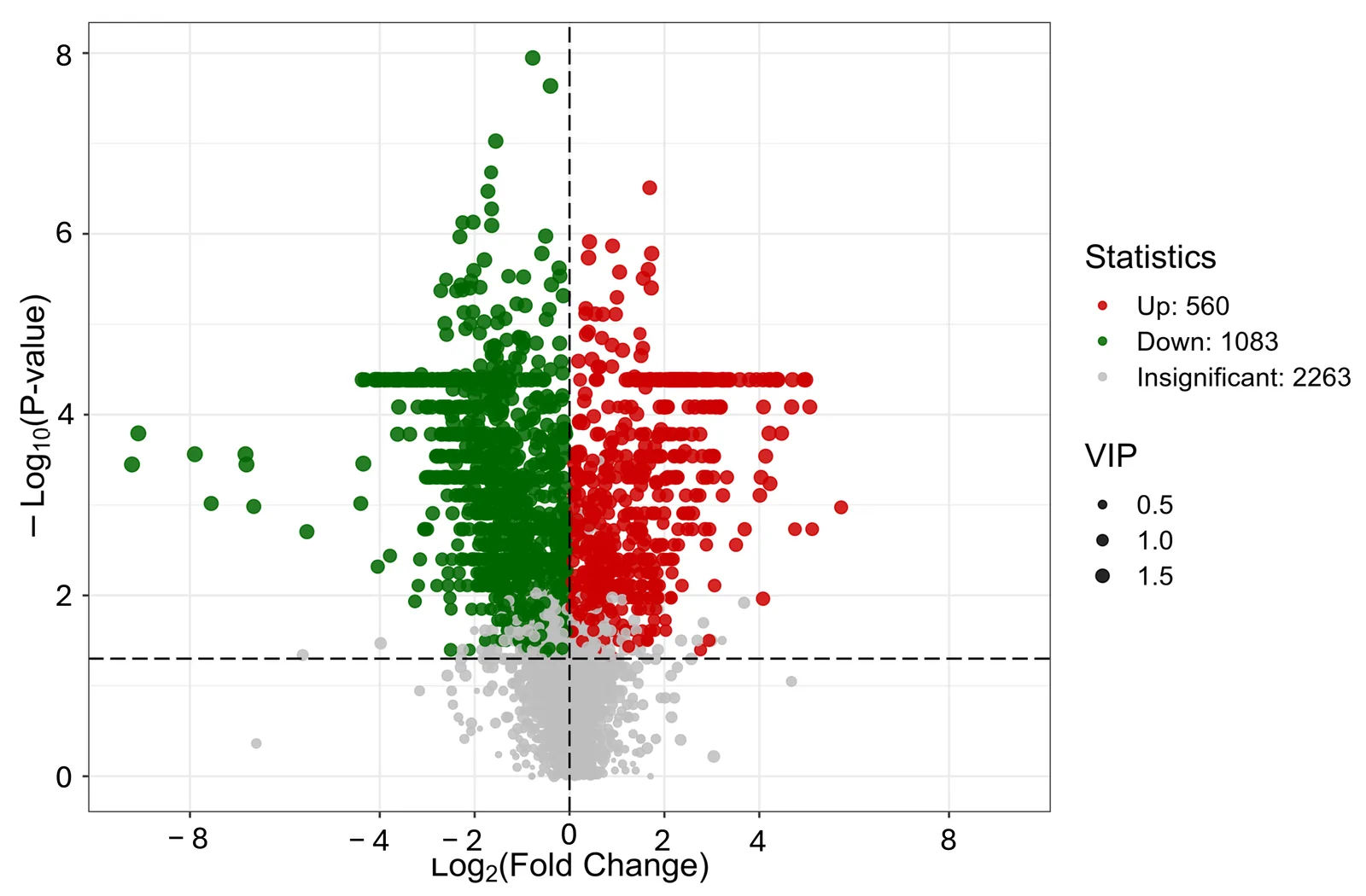
Volcano plot of differential metabolites between colostrum and mature milk in lactating sows. Red and green dots represent significantly increased or decreased metabolites (*p* < 0.05), respectively, while gray dots indicate non-significant metabolites.

**Figure 3:**
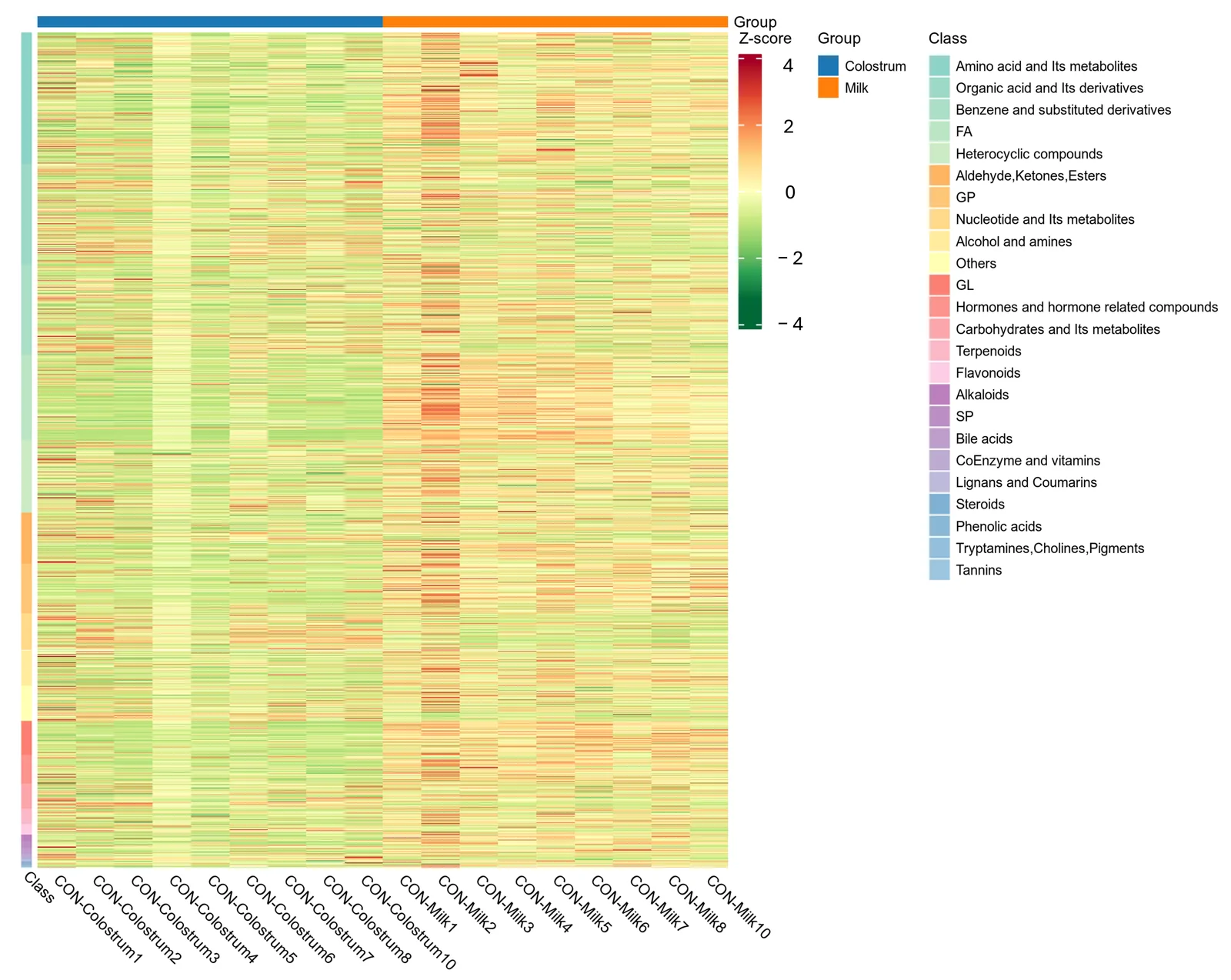
Heat map of differentially abundant metabolites between colostrum and mature milk in lactating sows.

A total of 3906 metabolites were putatively annotated based on spectral matching, database searches, and tandem mass spectrometry (MS2) profiles, and only these annotated metabolites were utilized for subsequent downstream analyses. Of these annotated metabolites, 560 were significantly increased, 1083 were decreased, and 2263 exhibited no statistical differences in the colostrum versus mature milk comparison. The detailed chemical classification of these metabolites is presented in Table 3, while their overall distribution and statistical significance are illustrated in the volcano plot (Figure 2) and heat map (Figure 3).

### 3.3. Multivariate Statistical Analysis

**Figure 4:**
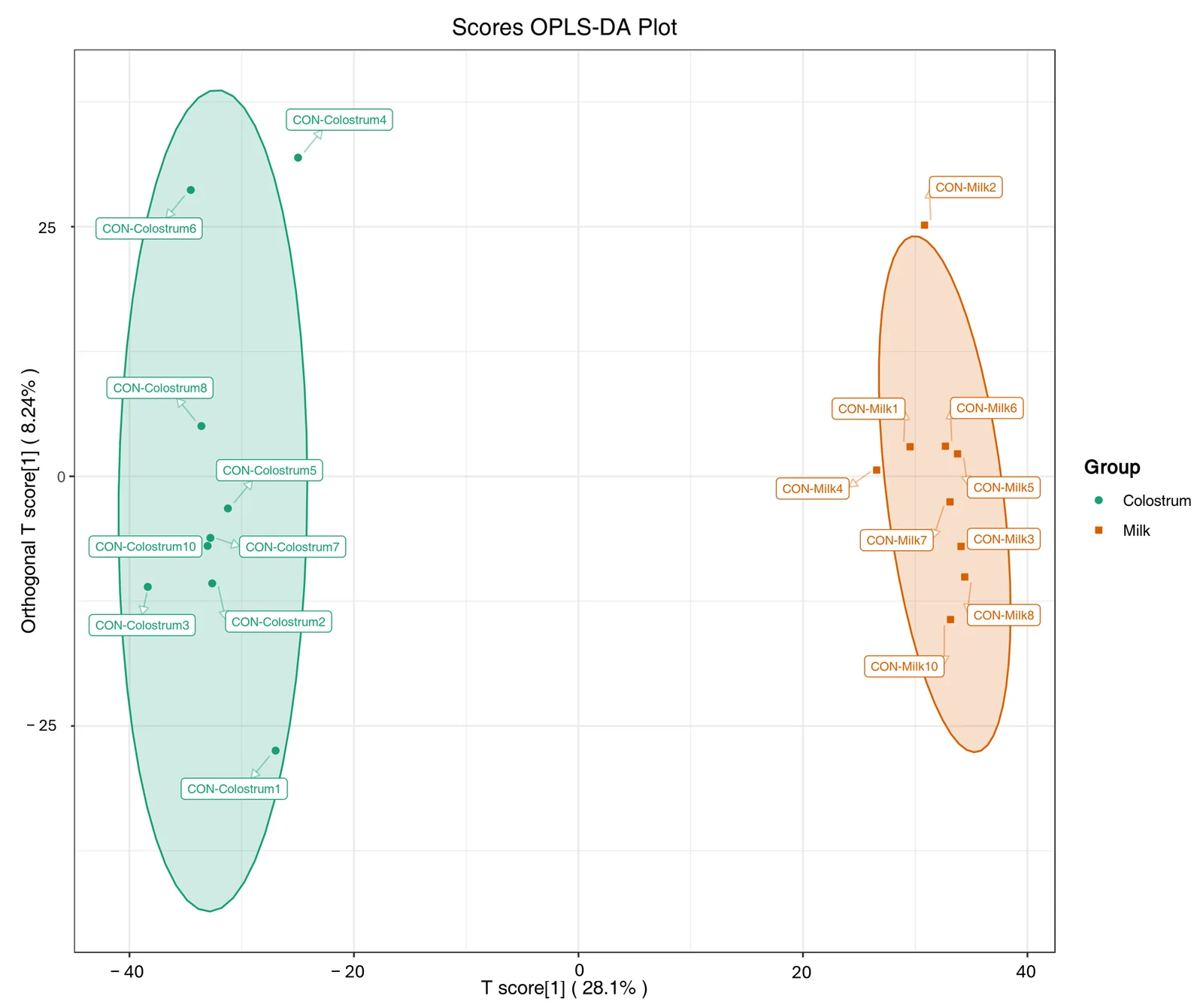
Orthogonal partial least squares–discriminant analysis (OPLS-DA) score plot of colostrum and mature milk in lactating sows. Each point represents an individual sample, and colors indicate the sample groups.

**Figure 5:**
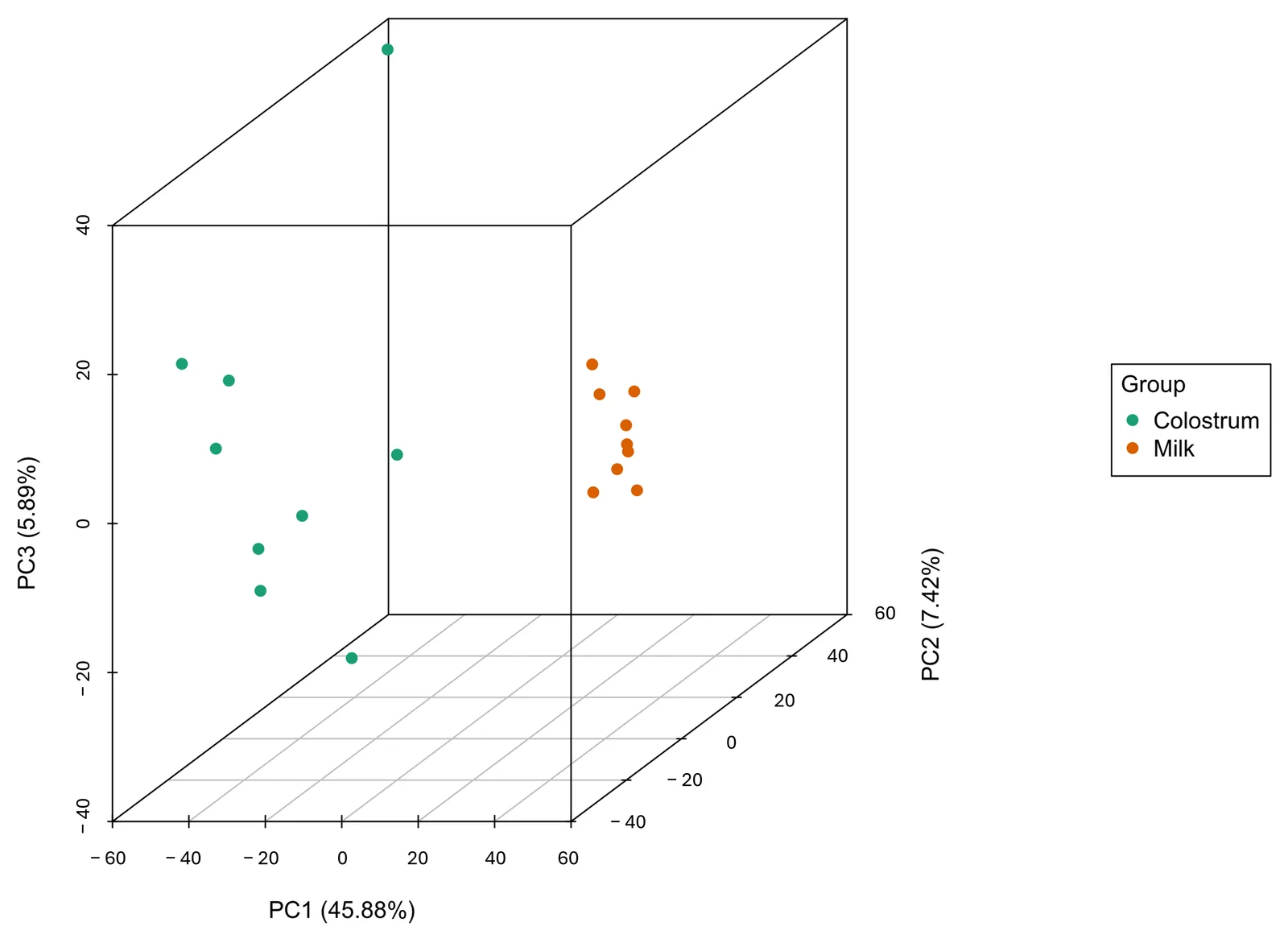
Three-dimensional principal component analysis (3D PCA) score plot of colostrum and mature milk in lactating sows. Each point represents an individual sample, and colors indicate the sample groups. PC1, PC2, and PC3 represent the first, second, and third principal components, respectively. The percentages associated with each principal component indicate the proportion of total variance explained by that component.

**Figure 6:**
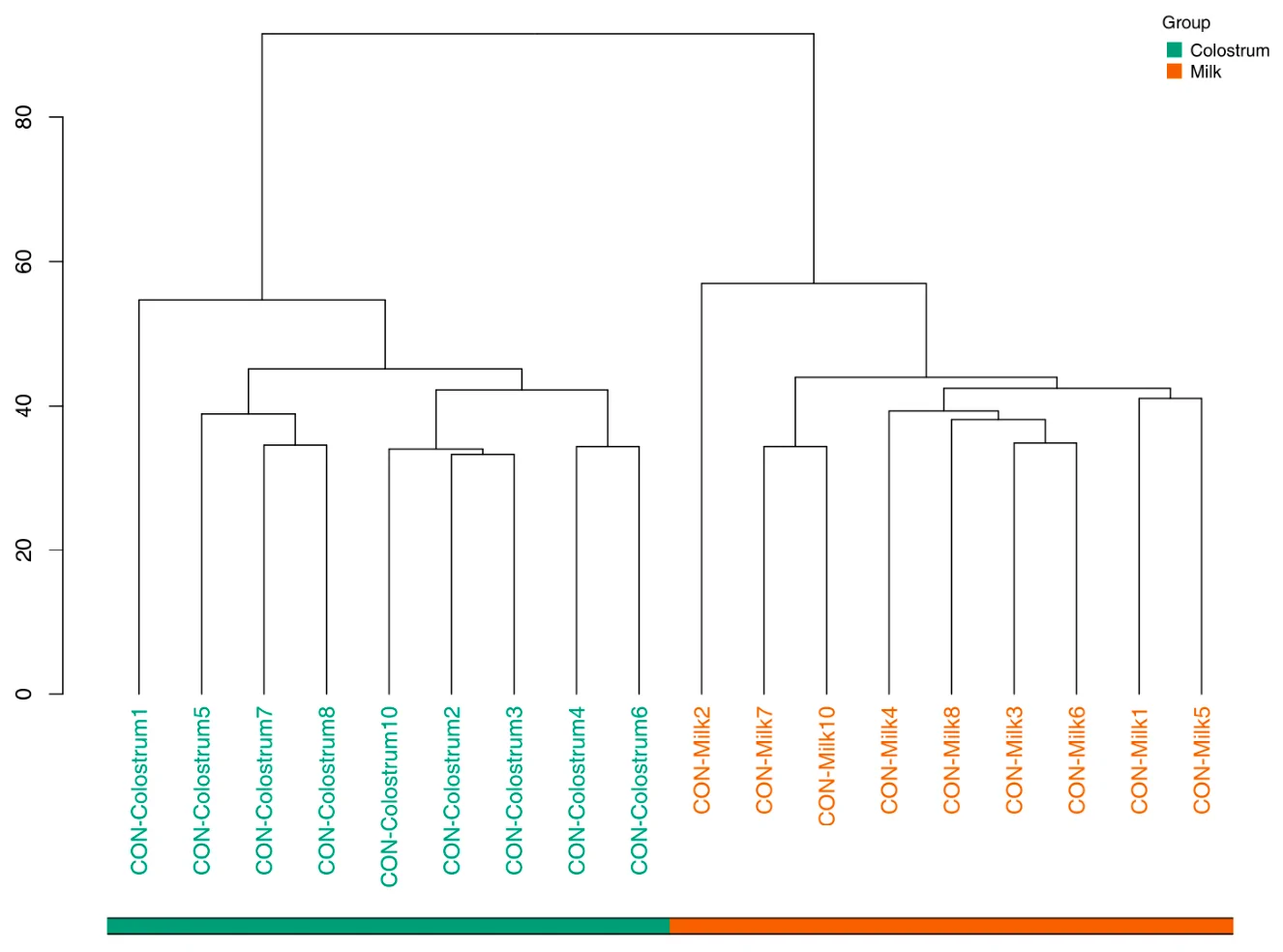
Hierarchical clustering tree (dendrogram) of colostrum and mature milk in lactating sows based on metabolomic profiles.

OPLS-DA model suggested strong performance with an R^2^Y (goodness of fit) value exceeding 0.99 and a cumulative Q^2^ (predictive ability) of 0.93 for the colostrum–mature milk comparison, indicating excellent model robustness. In general, Q^2^ values > 0.5 are considered indicative of an effective model, whereas values > 0.9 represent excellent predictive performance. OPLS-DA score plot suggested a distinct and complete separation between the metabolic profiles of colostrum and mature milk (Figure 4). Notably, the colostrum samples were more widely dispersed across the plot compared to the tightly clustered mature milk samples, indicating a substantially higher degree of individual variation in the metabolome during the colostrum phase. This clear separation was further validated by the 3D PCA model (Figure 5), where PC1, PC2, and PC3 accounted for 45.88%, 7.42%, and 5.89% of the total variance, respectively. Consistent with the OPLS-DA results, the 3D PCA plot showed well-separated clusters with colostrum samples exhibiting greater dispersion. Furthermore, hierarchical clustering tree analysis confirmed a complete and comprehensive segregation between the colostrum and mature milk metabolomes (Figure 6).

### 3.4. Top 15 Increased and Decreased Differential Metabolites in Colostrum Compared to Mature Milk

**Figure 7:**
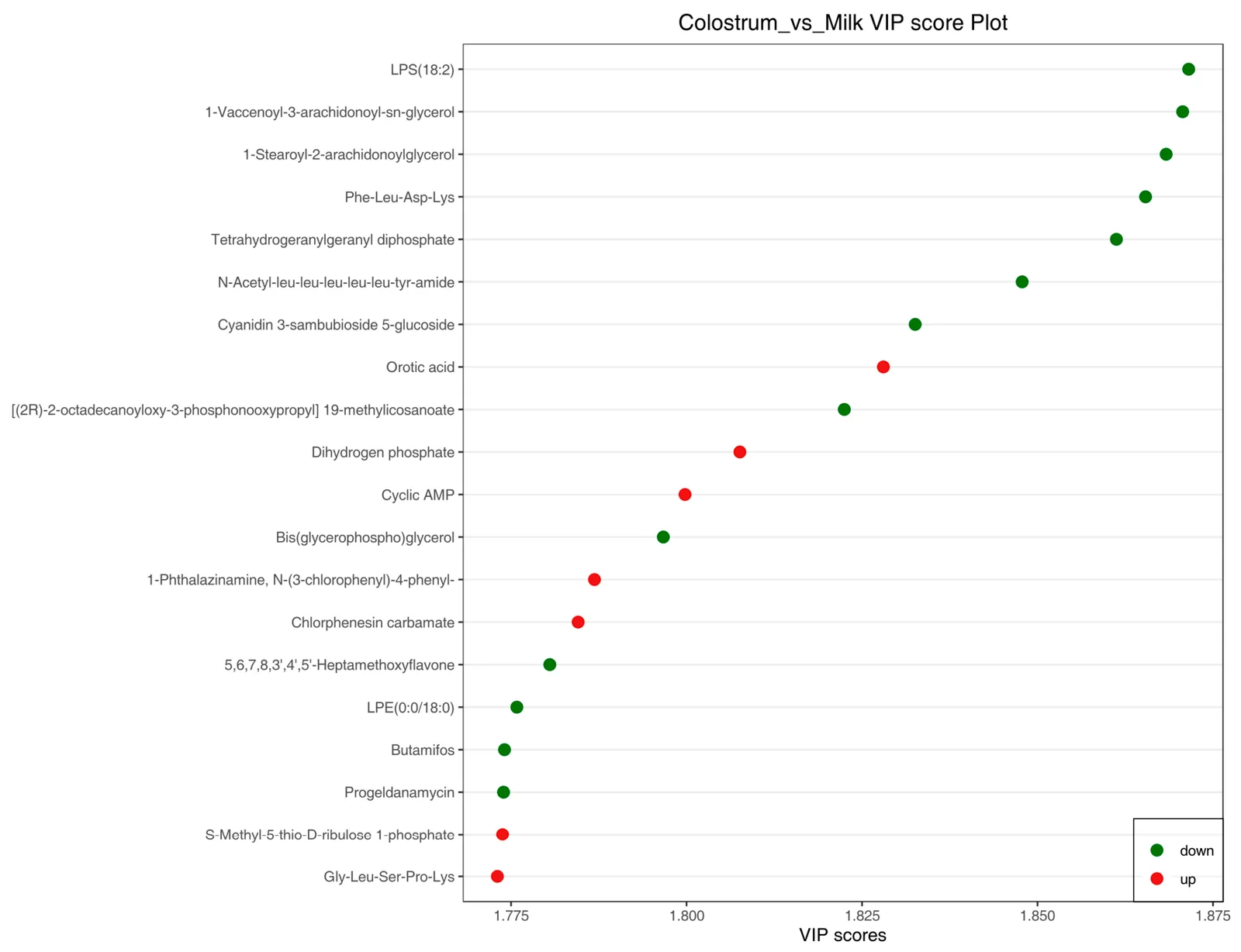
Metabolites with the highest variable importance in projection (VIP) scores distinguishing colostrum and mature milk in lactating sows including lipids, glycerol derivatives, peptides contributing to group separation.

Untargeted metabolomic analysis revealed distinct metabolic profiles between colostrum and mature milk. The top 15 significantly altered metabolites (*p* < 0.05) were classified as increased or decreased based on log2 fold change (Table 4). Increased metabolites in colostrum exhibited log2 fold changes ranging from 4.22 to 5.73. These included riboflavin (vitamin B2), orotic acid, and hydrogen phosphate. AICA ribonucleotide was also increased in colostrum. Steroid-related and plant-derived metabolites were detected among the increased group, including 16α-hydroxypregnenolone, asiaticoside A, and ginsenoside Ro. In addition, xenobiotic compounds such as chlorobenzilate and fenaminosulf were detected at higher levels in colostrum. Metabolites enriched in mature milk showed log2 fold changes ranging from 4.05 to 9.22. These compounds were mainly lipid-related metabolites including diacylglycerols and glycerolipids such as 1-vaccenoyl-3-arachidonoyl-sn-glycerol, 1,2-dioleoyl-rac-glycerol, 1-stearoyl-2-arachidonoylglycerol, 1,2-dioleoyl-sn-glycerol, and 1-palmitoyl-3-vaccenoyl-sn-glycerol. Additional lipid species including lysophosphatidylserine (LPS 18:2), lysophosphatidylethanolamine (LPE 16:1/0:0), and 9-decenoic acid were also increased in milk. Peptides and biosynthetic intermediates such as N-acetyl-leu-leu-leu-leu-leu-tyr-amide, Phe-Leu-Asp-Lys, tetrahydrogeranylgeranyl diphosphate, and 5″-phosphoribosylparomamine were also observed in this group. Figure 7 showed metabolites with the highest VIP scores including lipids, glycerol derivatives, and peptides, which contributed to the clear separation between colostrum and mature milk.

### 3.5. KEGG Pathway Enrichment Analysis of Differential Metabolites Between Colostrum and Mature Milk

Table 5 presents the KEGG pathway enrichment analysis of the differential metabolites identified between colostrum and mature milk. The analysis revealed profound alterations in several primary pathways with an overwhelming predominance directly governing lipid homeostasis and structural remodeling. Notably, lipid metabolism pathways—including glycerophospholipid, glycerolipid, arachidonic acid, and linoleic acid metabolism—were highly enriched with significant differential metabolites. Furthermore, pathway screening annotated critical physiological systems governing maternal nutrient delivery such as vitamin digestion and absorption as well as the biosynthesis of cofactors. Pathways associated with cellular membranes, intercellular signaling, and tissue remodeling were also highly represented including choline metabolism, glycosylphosphatidylinositol (GPI)-anchor biosynthesis, and inositol phosphate metabolism.

**Figure 8:**
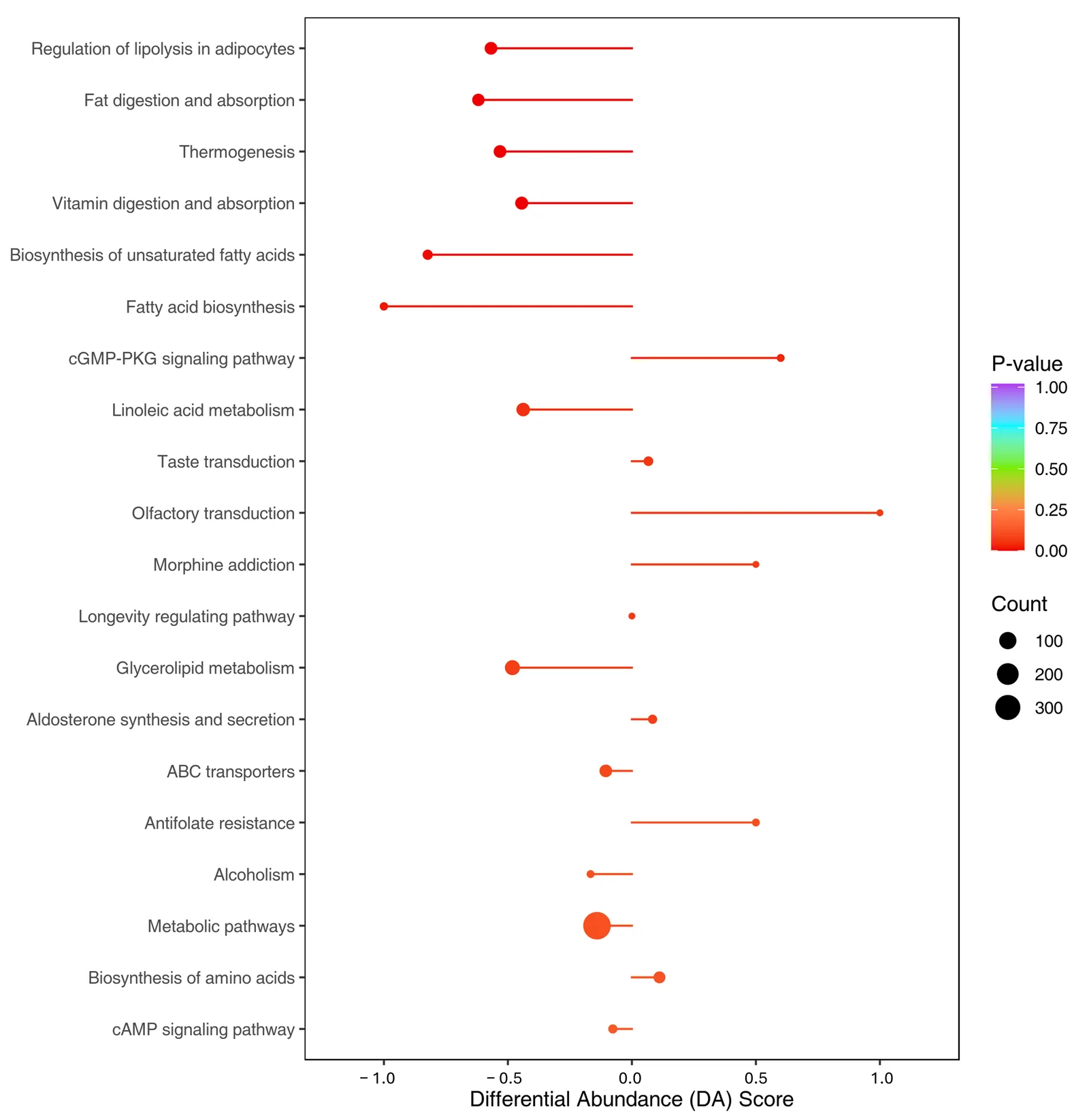
KEGG-based differential abundance (DA) score analysis between colostrum and mature milk. DA score was calculated as (number of increased metabolites—number of decreased metabolites) divided by the total number of annotated metabolites within each pathway with comparisons defined as colostrum relative to mature milk. Point size represents the total number of annotated metabolites within the corresponding pathway.

KEGG pathway enrichment analysis based on differential abundance (DA) scores (Figure 8) revealed profound metabolic alterations between colostrum and mature milk, with an overwhelming predominance of pathways associated with lipid homeostasis and structural remodeling (*p* < 0.05). Notably, key lipid metabolism pathways—including the regulation of lipolysis in adipocytes, fat digestion and absorption, fatty acid biosynthesis, glycerolipid metabolism, and linoleic acid metabolism—exhibited marked shifts, underscoring substantial maternal nutrient adaptation across lactation stages. Furthermore, pathway screening identified critical physiological systems reflecting maternal nutrient delivery and systemic metabolism, such as vitamin digestion and absorption, thermogenesis, and cofactor biosynthesis. Pathways associated with cellular membrane signaling and metabolic regulation, including the cGMP-PKG and cAMP signaling pathways, as well as ABC transporters, were also significantly represented. Overall, DA score analysis reflected that primary pathways characterized by energy mobilization, fat synthesis, and vitamin processing showed distinct compositional variations to support the evolving nutritional demands from early colostrum to established mature milk.

### 3.6. Molecular Weight and Log P (Log of Partition Coefficient) of Increased and Decreased Differential Metabolites

**Figure 9:**
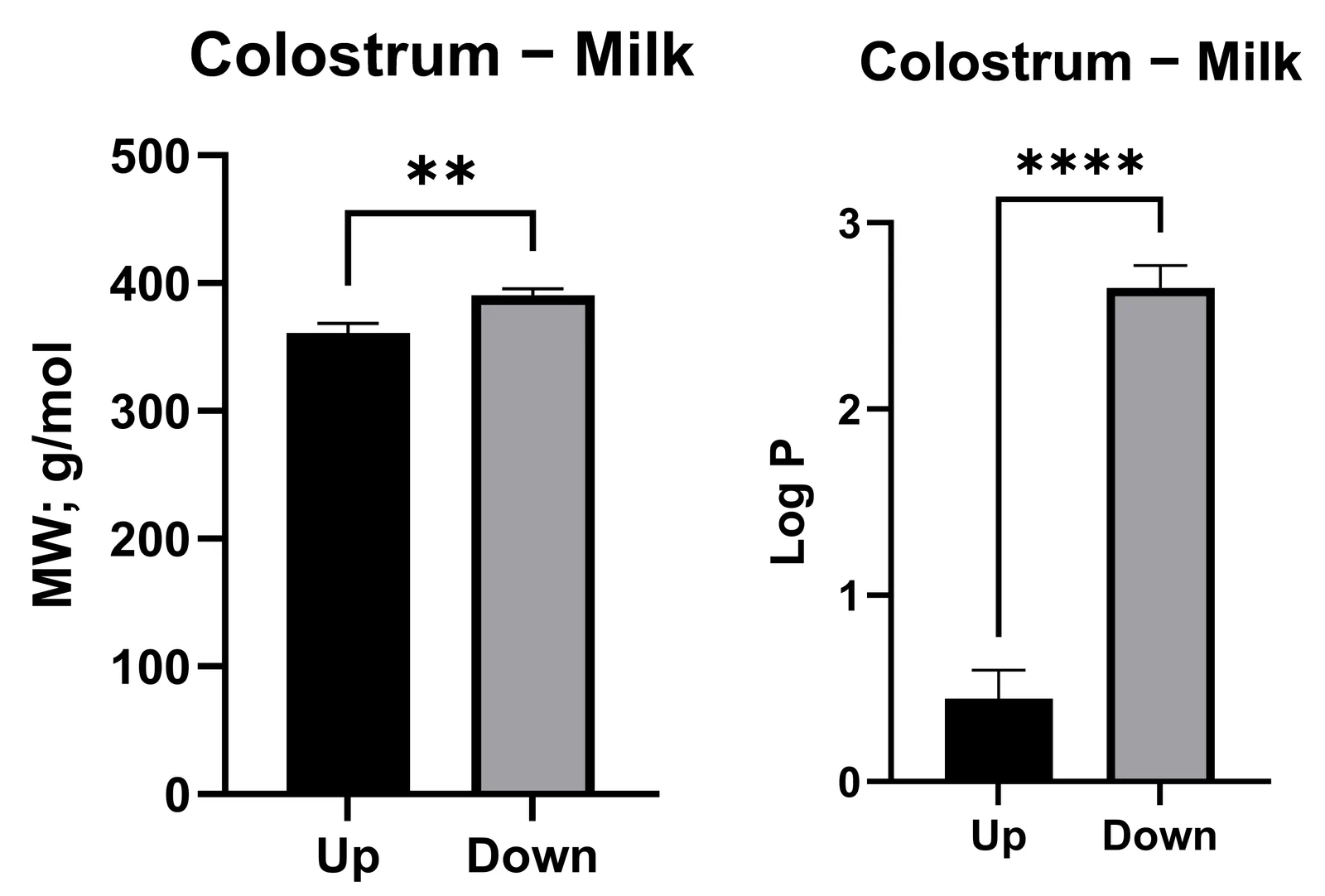
Molecular weight and Log P (logarithm of the octanol–water partition coefficient) of increased and decreased differential metabolites in the comparison between colostrum and mature milk. ** *p* < 0.01 and **** *p* < 0.0001. For molecular weight (MW), colostrum: 360.9 g/mol (SEM 9.3), milk: 390.5 g/mol (SEM: 9.3; *p* = 0.0016). For Log P, colostrum: 0.45, milk: 2.6 (SEM: 0.19; *p* < 0.0001). Values represent means ± SEM.

The molecular weight and octanol–water partition coefficient (Log P) of the differentially expressed metabolites between colostrum and mature milk are shown in Figure 9. Metabolites decreased in colostrum (i.e., those enriched in mature milk) exhibited significantly greater molecular weights (*p* < 0.01) and higher Log P (*p* < 0.001) values compared to those enriched in colostrum.

## 4. Discussion

The lactation period is a critical phase in swine production, directly affecting both sow physiological status and piglet growth and survival [20]. During this period, mammary secretions undergo substantial biological changes to meet the evolving nutritional and immunological needs of the offspring [21]. Therefore, a clear understanding of the biological and compositional differences between colostrum and mature milk is essential for optimizing lactational performance and neonatal outcomes. The objective of this study was to investigate the metabolic differences between colostrum and mature milk using an untargeted metabolomics approach. This approach enables a comprehensive characterization of metabolic profiles, providing insight into the physiological distinctions between colostrum and mature milk. In addition, identifying these differences may help inform strategies to improve the functional quality of lactational secretions and optimize piglet performance.

In the present study, milk samples were collected at two distinct lactational stages between 12 and 24 h post-farrowing (colostrum) and at d 28 of lactation (mature milk) from sows with an average parity of 5.1. Samples obtained between 12 and 24 h postpartum were classified as colostrum, consistent with established lactation staging criteria [6], whereas d 28 samples represented mature milk. In the US, weaning commonly occurs between 18 and 21 days of age; however, a 28-day weaning period is also widely practiced in other production systems and regions (e.g., European Union) for welfare and production reasons [22]. For this experiment, this extended lactation window allows for a more pronounced physiological distinction between early and late lactation. Accordingly, the present design captures the marked metabolic divergence between colostrum and mature milk, reflecting substantial shifts in maternal nutrient allocation and mammary gland metabolism. A previous study by Settachaimongkon et al. [14] investigated differences in diverse lipid profiles across lactation days (d 0, d 3, and d 17) and parities (parities 1, 2–6, and 7). Although parity did not exert a significant effect, marked lipidomic shifts were observed among d 0, d 3, and d 17. However, the study was restricted to early and mid-lactation, leaving the comprehensive metabolic transitions extending to fully mature milk at d 28 in high-parity sows (mean parity 5.1) largely uncharacterized. Furthermore, high-resolution untargeted profiling was necessary to capture subtle functional metabolite alterations that traditional targeted approaches might oversee. While the sample size was limited to nine sows per group, the OPLS-DA model yielded extremely high R^2^Y (>0.99) and Q^2^ (0.93) values, reflecting the profound and distinct metabolic shifts between colostrum and mature milk. Nonetheless, validation with larger cohorts in future studies is warranted to further confirm these robust metabolic variations. Although colostrum samples were collected within a strict 24-h window postpartum, slight variations in the exact timing of collection relative to farrowing can still introduce individual metabolic differences. Conversely, mature milk is collected at a more uniform and stable stage of established lactation, which may reflect the tighter clustering and lower inter-individual variation observed in the OPLS-DA score plot (Figure 4) and 3D PCA (Figure 5). Nevertheless, the inclusion of nine biological replicates (n=9) per group effectively mitigated this time-dependent variability, providing sufficient statistical power to establish a reliable metabolic baseline for early colostrum.

In the present study, proximate analysis revealed that protein content was significantly higher in colostrum compared with mature milk. Fat content did not differ statistically (numerically higher in colostrum; *p* = 0.25). A previous study by Zhang et al. [23] reported a consistent pattern across lactation, where protein content gradually decreased from colostrum to mature milk, whereas fat content tended to increase over time. Theil et al. [24] observed higher protein and lower fat concentrations in colostrum relative to later lactation stages. In addition, another study [25] reported an approximately three-fold higher protein concentration in colostrum compared with mature milk collected at d 60 postpartum, highlighting substantial temporal shifts in milk composition. There is a general consensus that colostrum contains higher protein levels than mature milk, largely reflecting its biological role in transferring essential functional proteins including immunoglobulins (e.g., IgG) to the neonate [6]. However, reported fat dynamics are more variable across studies, likely reflecting differences in sampling time points, lactation stage definition, and environmental or management conditions.

In contrast to the proximate analysis, which showed a numerical decrease in fat content in mature milk compared with colostrum, untargeted metabolomic analysis revealed elevated relative signals of lipid-related features during lactation in the current study. Specifically, 150 fatty acids were increased and 18 were decreased in mature milk, while 79 glycerophospholipids and 63 glycerolipids were increased, compared with only 18 glycerophospholipids and 4 glycerolipids metabolites that were decreased. These findings suggest that although the overall quantity of fat may not increase, the diversity and complexity of lipid species are markedly enhanced in mature milk. This is further supported by our metabolomic profiling (Figure 9), which suggests that differential metabolites enriched in mature milk exhibited significantly greater molecular weights and higher Log P values. Log P (the logarithm of the octanol–water partition coefficient) measures a molecule’s lipophilicity and partitioning behavior, thereby reflecting a shift in chemical-class composition toward more hydrophobic species in mature milk. These data imply that these compositional differences may stem, in part, from changes in mammary gland permeability during the transition from colostrogenesis to established lactation [26]. Lipids play critical roles in neonatal piglets, serving not only as a major energy source but also as essential components for cellular growth, membrane synthesis, thermoregulation, and physiological development [27,28]. Therefore, the observed shift in lipid composition may reflect changing nutritional requirements throughout lactation. Colostrum may primarily provide readily available energy-rich lipids such as triacylglycerols (TAG), to support the immediate metabolic demands of newborn piglets after birth. In contrast, mature milk appears to contain a broader spectrum of lipid molecules including fatty acids, glycerophospholipids, and glycerolipids, which may reflect enhanced nutritional provisions associated with ongoing tissue development, thermoregulation, organ maturation, and cellular function in growing piglets. Consistent with our findings, Zhao et al. [29] reported that high-molecular-weight unsaturated TAG including TAG 18:1/18:1/16:0 are enriched in colostrum, whereas mature milk contains greater proportions of low- and medium-molecular-weight TAGs and medium-chain fatty acids in humans. Furthermore, several long-chain fatty acids enriched in colostrum including linoleic acid (C18:2) and stearic acid (C18:0), have been reported to be positively associated with piglet body weight and survivability, highlighting the importance of colostrum-derived lipids in supporting neonatal growth and survival [30]. Ren et al. [31] also suggested distinct fatty acid distributions and triacylglycerol profiles between sow colostrum and mature milk. In their study, a total of 51 TAG species, including their isomers, differed significantly among sow colostrum, mature milk, and piglet formulas. Moreover, the present study indicates that diverse glycerolipids exhibited the highest VIP scores, suggesting that these molecules play a key role in discriminating between colostrum and mature milk. This apparent discrepancy can be reconciled by distinguishing total lipid mass (absolute concentration) from molecular species diversity (compositional complexity); while proximate analysis measures overall fat quantity, untargeted metabolomics captures a broader spectrum of distinct functional lipid species in mature milk, suggesting greater compositional diversification rather than directly demonstrating de novo lipogenesis or structural remodeling in the mammary gland. These findings support the fact that lipid composition, particularly TAG species distribution, is a key characteristic distinguishing colostrum from mature milk.

Similar to the lipid profiles between colostrum and mature milk, distinct temporal trends were observed in amino acid-related metabolite features in comparison between colostrum and mature milk. In the present study, protein levels were significantly greater in colostrum than in mature milk. This enrichment is consistent with the established physiological role of colostrum as a rich source of macromolecular proteins including caseins that support neonatal nutrition and growth, as well as bioactive proteins such as lactoferrin and maternal immunoglobulins that facilitate passive immune transfer and early-life protection [6]. Interestingly, at the untargeted metabolomic level, amino acid-related features exhibited a distinct profile, with 69 features detected at lower relative levels and 189 features detected at higher relative levels in mature milk compared to colostrum. This apparent discrepancy can be reconciled by distinguishing total protein and amino acid mass from molecular species diversity; while proximate and conventional analyses measure overall protein quantity, untargeted metabolomics captures a broader spectrum of distinct nitrogenous and amino acid-related species in mature milk, suggesting greater compositional diversification and metabolic dynamics rather than directly demonstrating enhanced free amino acid turnover or structural remodeling in the mammary gland. Furthermore, this distinction reflects a shift in metabolic profiles between the two lactation stages. Colostrum is characterized by lower-molecular-weight nitrogenous fractions, whereas mature milk exhibits a higher proportion of metabolites with greater molecular weights and higher Log P values (Figure 9), reflecting differences in physicochemical characteristics and relative feature abundances across lactation stages. Previous research suggested that although mature milk contained lower total concentrations of amino acids than colostrum, its amino acid profile was substantially altered across lactation [32,33]. These findings are consistent with our results and may help contextualize the chemical-class shifts observed between colostrum and mature milk. However, it is noteworthy that amino acids and low-molecular-weight peptides accounted for a smaller proportion among the top 15 differentially abundant metabolites, highest VIP scores, and top-tier enriched KEGG pathways in this untargeted model. While functional amino acid and peptide profiles undergo targeted alterations, lipid-related features prominently characterized the primary metabolic divergence between colostrum and mature milk in the present profiling. The localized shifts in specific functional peptides warrant further targeted investigation to fully elucidate their precise bioactive roles, alongside the broader lipid remodeling observed across lactation stages.

From a practical perspective, strategies aimed at modulating milk lipid composition may offer prospective opportunities to support maternal metabolic health and colostrum quality. Potential maternal approaches include optimizing sow lipid metabolism during late gestation and lactation or supplementing sow diets with targeted fat sources [34,35,36]. While previous studies have shown that dietary fat sources influence sow performance, most focused primarily on crude fat or selected lipid classes rather than offering a comprehensive profile. A deeper understanding of how specific lipid components shift across lactation stages may help guide future targeted nutritional strategies to support sow and piglet health.

In the present study, diverse xenobiotics such as pharmaceutical, industrial, and pesticide-related features were putatively annotated across both colostrum and mature milk in sows. While untargeted metabolomics serves as a powerful discovery tool, these findings must be interpreted with caution as tentative candidates rather than definitive absolute identifications, as they lack authentic standard confirmation. Nonetheless, the concurrent detection of these interrelated features strongly aligns with the previous literature demonstrating that various chemical contaminants are commonly detected in sow, cow, and human milks [37,38,39,40]. Future targeted validation studies utilizing definitive protocols are warranted to precisely establish absolute concentrations and exposure kinetics.

In the KEGG pathway analysis of the present study, an over-representation of nucleotide-related metabolites was observed across the Olfactory transduction, Morphine addiction, and Antifolate resistance pathways with colostrum exhibiting high enrichment scores for these specific categories. This downstream mismatching is primarily attributed to the high biological ubiquity and multi-pathway involvement of common metabolites such as cyclic adenosine monophosphate (cAMP), cyclic guanosine monophosphate (cGMP), adenosine monophosphate (AMP), and guanosine monophosphate (GMP) [41]. Biologically, the elevated presence of these precursors reflects the rich abundance of dietary nucleotides in colostrum, which functions to provision critical building blocks for DNA and RNA synthesis to support rapid cellular proliferation and gastrointestinal development in newborns [8,42]. However, the identification of pathways such as GPCR signaling, olfactory transduction, or antifolate resistance in the KEGG output primarily reflects the broad presence of ubiquitous nucleotide precursors across multiple database pathways rather than physiological shifts in these specific systems. Because these common metabolites participate in diverse metabolic networks, their automated cross-referencing naturally produces overlapping pathway annotations. Consequently, these specific pathway outputs should be viewed as inherent limitations of pathway mapping databases rather than true metabolic alterations.

## 5. Conclusions

This study provides a comprehensive characterization of the metabolic differences between colostrum and mature milk in sows. Clear compositional distinctions were revealed between the two lactation stages, reflecting a programmed structural divergence: annotated metabolite profiling suggested that colostrum is characterized by lower-molecular-weight and less processed nitrogenous fractions aligned with early neonatal metabolic demands, whereas mature milk exhibits higher-molecular-weight, more complex, and more lipophilic structures corresponding to shifting nutritional needs during later lactation. Importantly, the observed metabolic distinction between the two stages was predominantly driven by lipid-related compounds and lipidomic remodeling in the mammary gland. Nevertheless, a limitation of the present study is its reliance on untargeted metabolomics; thus, future targeted quantitative analyses and functional studies are warranted to validate specific key metabolites. Collectively, this study establishes a metabolic baseline of sow milk, highlighting the specific compositional distinctions that provide a foundation for advancements in sow nutrition and milk quality management.

## Figures and Tables

**Table 1:** Individual sow metadata, body weight dynamics, and litter performance characteristics ^1^.

Sow ID	Parity	Pre-Farrowing BW (kg)	Post-Lactation BW (kg)	Lactation Length (d)	Total Born	Live Born	Pre-Weaning Mortality (%)	Weaning BW (kg)
C1	4	289.8	274.4	27	16	9	0.0	6.6
C2	5	250.4	224.5	28	19	13	0.0	7.3
C3	5	301.6	283.5	27	20	16	23.1	5.2
C4	3	270.3	249.5	24	24	17	11.8	5.9
C5	6	267.6	247.2	24	10	10	0.0	8.3
C6	8	274.0	283.9	24	12	12	16.7	7.8
C7	5	262.2	207.3	25	18	17	8.3	6.7
C8	7	256.7	242.2	26	20	16	8.3	6.9
C10	3	300.0	239.5	27	13	13	8.3	8.4
Average	5.1	274.7	250.2	25.8	16.9	13.7	8.5	7.0
SD	1.7	18.5	26.3	1.6	4.5	3.0	8.0	1.0

^1^ All sows remained clinically healthy without any medication history throughout the trial period. Pre-weaning mortality (%) was calculated between d 1 and d 28 postpartum.

**Table 2:** Ingredient and calculated nutrient composition of diets for gestating and lactating sows (as-fed basis).

Items	Gestation, %	Lactation, %
Ingredients		
Corn, grain	72.55	61.73
Soybean meal, 45% CP	13.45	27
Distillers dried grains with solubles (DDGSs)	5	2
Soybean hulls	4	2
Soybean oil	1.8	3.75
Dicalcium phosphate	1.17	1.31
Limestone	0.62	0.73
DSM Vitamin premix ^1^	0.5	0.5
Common salt	0.5	0.5
Mineral premix ^2^	0.25	0.25
Choline chloride	0.15	0.15
L-Lysine HCl 78% ^3^	—	0.07
Ethoxyquin	0.01	0.01
Total	100	100
Calculated nutrient levels		
Metabolizable energy, Mcal/kg	3.26	3.4
CP, %	13.68	17
Calcium, %	0.69	0.8
Available phosphorus, %	0.35	0.4
Standardized ileal digestible (SID) Lysine, %	0.53	0.9

^1^ Provided per kilogram of complete diet: vitamin A, 10,472 IU; vitamin D3, 3307 IU; vitamin E, 88.2 IU; menadione, 6.61 mg; thiamine, 16.53 mg; riboflavin, 16.53 mg; pyridoxine, 16.53 mg; vitamin B12, 0.066 mg; niacin, 99.21 mg; pantothenic acid, 55.11 mg; folic acid, 16.53 mg; biotin, 2.20 mg (Dsm-firmenich, Parsippany, NJ, USA). ^2^ Provided per kilogram of complete diet: Mn, 55.0 mg; Fe, 180.0 mg; Cu, 27.5 mg; Zn, 182.5 mg; Se, 0.466 mg; I, 0.495 mg (Pinnacle Premix, Hereford, TX, USA). ^3^ L-Lysine HCl (Best Amino; 99% purity, providing 78.8% active L-lysine; CJ Bio America, Downers Grove, IL, USA).

**Table 3:** Chemical classification and distribution of putatively annotated metabolites in sow colostrum and mature milk using untargeted metabolomics.

Metabolite Class/Category	Total Putatively Annotated	Total Differential	Increased	Decreased
Benzene and substituted derivatives	576	178	77	101
Amino acids and their metabolites	635	258	69	189
Organic acids and their derivatives	439	198	103	95
Heterocyclic compounds	400	142	52	90
Aldehydes, ketones, and esters	281	101	35	66
Fatty acids	292	168	18	150
Alcohols and amines	174	70	21	49
Glycerophospholipids	186	97	18	79
Nucleotides and their metabolites	150	73	49	24
Glycerolipids	111	67	4	63
Hormones and hormone-related compounds	118	56	11	45
Carbohydrates and their metabolites	91	50	29	21
Terpenoids	64	30	8	22
Flavonoids	63	21	11	10
Alkaloids	47	13	2	11
Sphingolipids	24	13	4	9
Coenzymes and vitamins	28	10	7	3
Bile acids	27	12	3	9
Steroids	14	4	2	2
Lignans and coumarins	14	5	1	4
Tryptamines, choline compounds, and pigments	9	3	0	3
Phenolic acids	8	3	1	2
Tannins	2	2	0	2
Others	153	69	35	34
Total MS2 Putatively Annotated	3906	1643	560	1083

**Table 4:** Top 15 increased and decreased metabolites between colostrum and mature milk using untargeted metabolomics. Variable importance in projection (VIP) score indicates the contribution of each metabolite to group discrimination in the OPLS-DA model; metabolites with VIP > 1 were considered important contributors. Log2FC, log2 fold change.

**Order**	**Compounds (High in Colostrum)**	**VIP**	***p*** **Value**	**Log2FC**
1	16alpha-Hydroxypregnenolone	1.33	0.001	5.73
2	(1S,4aS,7S,7aR)-7-methyl-1-[(2S,3R,4S,5R,6R)-3,4,5-triacetoxy-6-(acetoxymethyl)tetrahydropyran-2-yl]oxy-1,4a,5,6,7,7a-hexahydrocyclopenta[c]pyran-4-carboxylic acid	1.29	0.002	5.11
3	AICA ribonucleotide	1.59	<0.001	5.07
4	Asiaticoside A	1.74	<0.001	4.97
5	Chlorobenzilate	1.59	<0.001	4.94
6	Fenaminosulf	1.29	0.002	4.75
7	Riboflavin	1.67	<0.001	4.70
8	Arabinonic acid	1.69	<0.001	4.68
9	Cadabicine	1.58	<0.001	4.47
10	Toyocamycin	1.74	<0.001	4.39
11	2-Hydroxy-3-(3,4,5-trimethoxyphenyl)propanoic acid	1.69	<0.001	4.37
12	Orotic acid	1.83	<0.001	4.36
13	Benzylsuccinyl-CoA	1.64	<0.001	4.34
14	Ginsenoside Ro	1.59	<0.001	4.23
15	Hydrogen phosphate	1.77	<0.001	4.22
**Order**	**Compounds (High in Mature Milk)**	**VIP**	***p*** **Value**	**Log2FC**
1	LPS(18:2)	1.87	<0.001	−9.22
2	N-Acetyl-leu-leu-leu-leu-leu-tyr-amide	1.85	<0.001	−9.09
3	1-Vaccenoyl-3-arachidonoyl-sn-glycerol	1.87	<0.001	−7.90
4	1,2-Dioleoyl-rac-glycerol	1.63	<0.001	−7.55
5	1-Stearoyl-2-arachidonoylglycerol	1.87	<0.001	−6.83
6	Phe-Leu-Asp-Lys	1.87	<0.001	−6.81
7	[(2S)-2-dodecanoyloxy-3-hydroxypropyl] 20-methylhenicosanoate	1.60	0.001	−6.66
8	1,2-Dioleoyl-sn-glycerol	1.64	0.002	−5.54
9	1-Palmitoyl-3-vaccenoyl-sn-glycerol	1.58	<0.001	−4.40
10	2,3-Dihydroxypropyl 2-[(octadec-9-enoyl)amino]ethyl hydrogen phosphate	1.73	<0.001	−4.36
11	Tetrahydrogeranylgeranyl diphosphate	1.86	<0.001	−4.35
12	5″-Phosphoribosylparomamine	1.62	<0.001	−4.31
13	[(2R)-2-octadecanoyloxy-3-phosphonooxypropyl] 19-methylicosanoate	1.82	<0.001	−4.10
14	9-Decenoic acid	1.69	<0.001	−4.06
15	LPE(16:1/0:0)	1.77	<0.001	−4.06

**Table 5:** Top 15 KEGG pathways based on metabolite annotation in colostrum and mature milk of lactating sows comparison using untargeted metabolomics.

Order	KEGG Pathway	Number of Differential Metabolites	Total Number of Metabolites
1	Metabolic pathways	386	753
2	Glycerophospholipid metabolism	120	228
3	Retrograde endocannabinoid signaling	73	140
4	Glycerolipid metabolism	63	110
5	Arachidonic acid metabolism	45	82
6	Linoleic acid metabolism	44	73
7	Biosynthesis of cofactors	42	86
8	Vitamin digestion and absorption	38	54
9	Choline metabolism in cancer	37	70
10	Kaposi sarcoma-associated herpesvirus infection	37	74
11	Glycosylphosphatidylinositol (GPI)-anchor biosynthesis	36	71
12	Regulation of lipolysis in adipocytes	35	44
13	Thermogenesis	35	47
14	Inositol phosphate metabolism	35	73
15	Long-term depression	35	73

## Data Availability

The raw dataset is available in Supplementary Table S1.

## Supplementary Materials

The following supporting information can be downloaded at: https://www.mdpi.com/article/10.3390/ani16213504/s1, Supplementary Table S1. Raw metabolomics dataset of sow colostrum and mature milk samples.

## Abbreviations

The following abbreviations are used in this manuscript:

DA	Differential Abundance
KEGG	Kyoto Encyclopedia of Genes and Genomes
OPLS-DA	Orthogonal Partial Least Squares–Discriminant Analysis
PCA	Principal Component Analysis
VIP	Variable Importance in Projection
